# DNA damage and senescence in the aging and Alzheimer’s disease cortex are not uniformly distributed

**DOI:** 10.1002/alz.095624

**Published:** 2025-01-09

**Authors:** Karl Herrup, Gnanesh Gutta, Jay Mehta, Rody Kingston, Fulin Ma

**Affiliations:** ^1^ University of Pittsburgh School of Medicine, Pittsburgh, PA USA; ^2^ University of Pittsburgh, Pittsburgh, PA USA; ^3^ University of Pittsburgh, School of Pharmacy, Pittsburgh, PA USA

## Abstract

**Background:**

Alzheimer’s disease (AD) has a typical age of onset exceeding 65 years. The age‐dependency of the condition led us to track the appearance of DNA damage in the frontal cortex of individuals who died with a diagnosis of AD. The focus on DNA damage was motivated by evidence that increasing levels of irreparable DNA damage are a major driver of the aging process. This connection between aging and genomic integrity is compelling because DNA damage has also been identified as a possible cause of cellular senescence. The number of senescent cells increase with ages, and their senescence‐associated secreted products likely contribute to age‐related illnesses.

**Method:**

Immunocytochemistry was performed on 12 µm sections of brain tissue from prefrontal cortex (BA9). We tracked DNA damage with 53BP1 or 𝛾‐H2AX and cellular senescence with p16 or p27 immunostaining. Embryonic mouse cortical neurons were cultured for 14 days in vitro before exposure to etoposide to induce DNA damage. Cell counts and labeling intensity were measured using QuPath.

**Result:**

DNA damage was significantly increased in the BA9 region of the AD cortex compared to the unaffected controls (UC). In the AD but not UC cases, the density of cells with DNA damage increased with distance from the pia mater up to approximately layer V then decreased in deeper areas. This pattern of DNA damage was overlaid with the pattern of cellular senescence, which also increased with cortical depth. On a cell‐by‐cell basis, we found that the intensity of the two markers was tightly linked in the AD, but not the UC brain. To test whether DNA damage was a causal factor in the emergence of senescence, we used etoposide treatment to damage the DNA of cultured mouse primary neurons but found no change in the expression of senescence‐associated markers.

**Conclusion:**

Our work suggests that DNA damage and cellular senescence are increased in the AD brain, and increasingly coupled. We propose that in vivo the relationship between the two age‐related processes is more complex than previously thought.

